# Composition and Biological Properties of Blanched Skin and Blanch Water Belonging to Three Sicilian Almond Cultivars

**DOI:** 10.3390/nu15061545

**Published:** 2023-03-22

**Authors:** Mariarosaria Ingegneri, Antonella Smeriglio, Rossana Rando, Teresa Gervasi, Maria Pia Tamburello, Giovanna Ginestra, Erminia La Camera, Rosamaria Pennisi, Maria Teresa Sciortino, Giuseppina Mandalari, Domenico Trombetta

**Affiliations:** 1 Department of Chemical, Biological, Pharmaceutical and Environmental Sciences, University of Messina, Viale Ferdinando Stagno d’Alcontres 31, 98166 Messina, Italy; 2 Department of Biomedical, Dental, Morphological and Functional Image Sciences (BIOMORF), University of Messina, Via Giovanni Palatucci, 98168 Messina, Italy

**Keywords:** almond by-products, cultivar, nutritional profile, polyphenols, antioxidant properties, prebiotic effect, antimicrobial activity, antiviral activity

## Abstract

The almond industry produces, by bleaching and stripping, two by-products: blanched skin (BS) and blanch water (BW). The aim of this study was to investigate the nutritional and polyphenolic profile, as well as the antioxidant, antimicrobial, antiviral, and potential prebiotic effects of BS and BW from three different Sicilian cultivars. The total phenols and flavonoids contents were ≥1.72 and ≥0.56 g gallic acid equivalents and ≥0.52 and ≥0.18 g rutin equivalents/100 g dry extract (DE) in BS and BW, respectively. The antioxidant activity, evaluated by 2,2-diphenyl-1-picrylhydrazyl scavenging ability, trolox equivalent antioxidant capacity, ferric-reducing antioxidant power, and oxygen radical absorbance capacity, was ≥3.07 and ≥0.83 g trolox equivalent/100 g DE in BS and BW, respectively. Isorhamnetin-3-*O*-glucoside was the most abundant flavonoid detected in both by-products. No antimicrobial effect was recorded, whereas BS samples exerted antiviral activity against herpes simplex virus 1 (EC_50_ 160.96 μg/mL). BS also showed high fibre (≥52.67%) and protein (≥10.99) contents and low fat (≤15.35%) and sugars (≤5.55%), making it nutritionally interesting. The present study proved that the cultivar is not a discriminating factor in determining the chemical and biological properties of BS and BW.

## 1. Introduction

Due to their exceptional nutritional, physico-chemical, and sensory features [1], almonds (*Prunus dulcis* Mill. DA Webb, also known by the synonym of *Prunus amygdalus* Batsch or *Amygdalus communis* L.) occupy first place within the world production of nuts [2,3]. They are considered a healthy and functional food [4,5] since they represent a reliable source of nutrients, including proteins, monounsaturated (MUFA) and polyunsaturated fatty acids (PUFA), vitamins, minerals, dietary fibres, and bioactive molecules with well-known antioxidant properties, such as polyphenols [1,6,7,8]. Numerous epidemiological and clinical studies have shown that regular consumption of almonds, as part of a balanced diet, is associated with beneficial and protective health effects closely related to nutrient and non-nutrient compounds [1,4,5], such as reduction in blood levels of total cholesterol and low-density lipoprotein (LDL) [8], modulation of serum glucose levels, improvement in intestinal microbiota profile, and protection against obesity, diabetes, metabolic syndrome, and cardiovascular diseases [4,9,10].

Therefore, a significant and constant increase in production worldwide [7] has been detected in recent years. According to Food and Agriculture Organization (FAO) reports, more than 3 million tons of almonds are produced each year [2]. Currently, the United States (California) is confirmed as the world leader in almond production (78%), followed by Australia, Spain, Turkey, and Italy (8%, 6%, 3%, and 2%, respectively) [6,11]. In this context, the preference for Italian almonds is constantly growing and cultivation of almonds in Sicily is experiencing a phase of great interest due to their unique aromatic taste [3]. Amongst the most widespread varieties, certainly Avola almonds are the best-known, with Pizzuta, Fascionello, and Romana cultivars growing in the provinces of Syracuse and Ragusa, the sunniest in Italy [12]. Another cultivar of notable interest is Tuono, originally from Puglia, but now widely cultivated in Sicily within the same areas.

Despite their beneficial effect, the increased production of almonds also proportionally implements the amount of processing by-products [7], mainly hulls (0.8–1.7 million tons/y) and shells (over 6 million tons/y) [2], which are normally discarded as special waste, involving considerable costs for manufacturing companies [2,3,5]. Given the large amount of biomass produced, companies have recently begun to evaluate adequate reuse of these by-products from a circular economy perspective in the energy and animal feed sector [5]. In order to meet the high market demand for peeled almonds, the almond industry produces, by means of a bleaching and stripping process [13,14], two by-products, which are much more interesting from a nutritional and health point of view [2]: blanched skin (BS) and blanch water (BW) [5]. BS, which represents 4–8% of the total shelled almond weight, is interesting both for high fibre and polyphenols content, the latter being concentrated in the almond skin [5,10,15]. BW is equally important as many of the water-soluble polyphenols are lost during the blanching process [5], so this matrix becomes an important source from which they can be recovered. Several studies have been undertaken on almond by-products, and our research group has already dealt with characterization and study of some biological activities of Californian and Sicilian BS and BW samples, including their antimicrobial and antiviral potential [5,13,15,16,17,18]. However, one of the major problems of the literature available in this context is that it is often not possible to trace the cultivar investigated; it is, therefore, not possible to relate a specific phytochemical profile, and, therefore, the nutritional and/or biological properties, to a specific cultivar. Furthermore, no study has ever compared Sicilian almond by-products from several cultivars to evaluate whether the cultivar effectively represents a discriminating factor in conferring certain phytochemical, nutritional, or biological properties to by-products.

The aim of the present study was to investigate, for the first time, the nutritional and phytochemical profile and antioxidant, antimicrobial, antiviral, and prebiotic potential of BS and BW samples from three different cultivars (Fascionello, Pizzuta, and Tuono) provided by various producers belonging to the Avola Almond Consortium, in order to evaluate potential reuse of these by-products in the Sicilian almond supply chain. Indeed, the knowledge of the compositional profile of these industrial by-products, which reflects the growth pedoclimatic conditions and cultivation and bleaching process, could be relevant for their potential application in the nutraceutical and pharmaceutical fields.

## 2. Materials and Methods

### 2.1. Chemicals

Folin–Ciocalteu, gallic acid, sodium carbonate (Na_2_CO_3_), sodium nitrite (NaNO_2_), aluminium chloride (AlCl_3_), sodium hydroxide (NaOH), 2,2-diphenyl-1-picrylhydrazyl (DPPH), 6-hydroxy-2,5,7,8-tetramethylchromane-2-carboxylic acid (trolox), potassium peroxydisulfate (K_2_S_2_O_8_), 2,2′-azino-bis (3-ethylbenzothiazoline-6-sulfonic acid) diammonium salt (ABTS), 2-4-6-tris(2-pyridyl)-s-triazine (TPTZ), sodium phosphate dibasic (Na_2_HPO_4_), potassium phosphate monobasic (KH_2_PO_4_), sodium acetate (CH_3_COONa), glacial acetic acid, iron(III) chloride hexahydrate (FeCl_3_·6H_2_O), iron(II) chloride tetrahydrate (FeCl_2_·4H_2_O), 1,1 2,2′-azobis(2-amidinopropane) dihydrochloride (AAPH), fluorescein disodium salt, 37 component fatty acid methyl ester (FAME) mix (certified reference material, TraceCERT^®^) were purchased from Merck (Darmstadt, Germany). The reference standards used for polyphenol characterization were purchased from Extrasynthese (Genay, France). Unless otherwise specified, chemicals and solvents were of analytical grade.

### 2.2. Sample Collection and Preparation

Thirteen samples of industrial almond blanched skin (BS) obtained from three different cultivars (6 Fascionello, 6 Pizzuta, and 1 Tuono) and their respective blanch water (BW) samples have been supplied by various producers within the Avola Almond Consortium. Producers dried the BS samples overnight at low temperature (30 °C); they were then powdered to obtain uniform particle size, whereas the BW samples were hermetically sealed in dark glass bottles and stored at 4 °C. Samples were shipped to the laboratory within 24 h from the blanching and stripping process and processed immediately.

BW samples were then brought to room temperature (RT), mixed, and subsequently dried (20 mL) at 37 °C in the dark using a rotary evaporator equipped with a high-performance vacuum pump capable of reaching a minimum of 5 mbar of pressure (Hei-VAP Core, Heidolph Instruments GmbH & Co., Schwabach, Germany). The residue was stored at +4 °C at most overnight and then suitably processed to extract the polyphenols, according to Smeriglio et al. [5]. Briefly, extraction was carried out by adding 100 mL methanol/HCl 0.1% (*v*/*v*) and sonicating for 10 min. The mixture was centrifuged (5000× *g*, 10 min, 4 °C) and the pellet extracted again. Supernatants were combined and dried, obtaining an average extraction yield of 0.30% (from 20 mL BW).

BS samples (10 g) were defatted with *n*-hexane (70 mL) for 6 h under stirring three times. The residue was mixed with 100 mL methanol/HCl 0.1% (*v*/*v*) and extracted thrice by sonication (15 min). Supernatants were combined and dried under the same conditions reported above. The pellet was dissolved in 40 mL deionized water and extracted four times with 40 mL ethyl acetate. The organic phases were combined, left 20 min on anhydrous sodium sulphate bed, and then dried by rotary evaporator, obtaining an average extraction yield of 1.64%.

The BS and BW dried extracts (BSE and BWE, respectively) were stored in a vacuum desiccator in the dark and freshly solubilized in methanol for phytochemical and in vitro cell-free tests, or in DMSO for cell-based assays, at the time of the analyses.

### 2.3. Nutritional Profile and Fatty Acids Composition of Blanched Skin

The nutritional profile of BS samples was evaluated on three independent samples in triplicate (*n* = 3). Energy, ash, proteins, fats, moisture, dietary fibres, and sugars were determined according to ISTISAN 1996/34 methods [19].

The fatty acid profile was evaluated according to Occhiuto et al. [20]. Briefly, BS samples were extracted with a chloroform/methanol (2:1, *v*/*v*) mixture thrice and centrifuged at 3000× *g* at RT. Supernatants were recovered and dried by nitrogen at RT. Transesterification with a 14% boron (III) fluoride methanol solution was carried out to obtain FAMEs, which were analysed by gas-chromatography coupled with flame ionization and mass spectrometry detectors (GC–FID and GC–MS, respectively) according to the elution program and setting parameters reported in Occhiuto et al. [20]. Detected compounds were identified based on the following parameters: matching of mass spectra with those reported in the MS library (NIST 08), comparison of MS fragmentation patterns with those reported in the literature, and co-injection with a Supelco 37 component FAME mix (see Section 2.1).

### 2.4. Phytochemical Screening

#### 2.4.1. Total Phenols

Total phenols were quantified according to Trombetta et al. [21] with some modifications. Briefly, 10 µL of BSE and BWE (0.31–2.5 mg/mL and 0.63–5 mg/mL) were added to 90 µL of deionized water and 100 µL Folin–Ciocalteu reagent. After 3 min incubation, 10% Na_2_CO_3_ (100 µL) was added. Samples were incubated in the dark at RT for 60 min, vortex-mixing every 10 min. Absorbance was read at 785 nm by using a UV–vis reader plate (Multiskan GO; Thermo Scientific, Waltham, MA, USA). Methanol was used as blank, whereas gallic acid as reference standard (0.075–0.6 mg/mL). Results were expressed as g gallic acid equivalents (GAE)/100 g dry extract (DE).

#### 2.4.2. Total Flavonoids

Total flavonoids were quantified according to Lenucci et al. [22]. Briefly, 50 µL of BSE and BWE (0.31–2.5 mg/mL and 0.63–5 mg/mL) were added to 450 µL of deionized water, followed by 30 µL of 5% NaNO_2_. After 5 min incubation at RT, 60 µL of 10% AlCl_3_ were added and samples incubated again for 6 min. Two-hundred microliters of 1 M NaOH and 210 µL of deionized water were added and vortex-mixed. The absorbance was recorded at 510 nm against methanol as blank. Rutin was used as reference standard (0.125–1.0 mg/mL) and results were expressed as g rutin equivalents (RE)/100 g DE.

### 2.5. Qualitative and Quantitative Analysis of Polyphenols by LC-DAD-ESI-MS

Polyphenols characterization of BSE and BWE was carried out by LC-DAD-ESI-MS analysis according to the elution program and setting parameters reported in Smeriglio et al. [23]. Identification of all the polyphenols was carried out by comparing their UV–vis spectra, retention times, and mass spectra with commercially available standards. Quantification was performed by building external standard calibration curves (for standard specification, see Section 2.1). The results were expressed as mg of each compound/100 g of DE.

### 2.6. Antioxidant Activity

#### 2.6.1. 2,2-Diphenyl-1-picrylhydrazyl (DPPH) Assay

The DPPH assay was carried out according to Smeriglio et al. [24] with some modifications. Briefly, 3.75 μL of BSE and BWE (15–120 µg/mL) were added to 150 µL fresh 1 mM DPPH methanol solution, mixed, and incubated in the dark for 20 min. The absorbance was recorded at 517 nm against methanol as blank and using the same instrument reported in Section 2.4.1. Trolox was used as reference compound (0.63–5.0 µg/mL). The results, which represent the average of three independent experiments in triplicate (*n* = 3), were expressed as g of trolox equivalents (TE)/100 g DE.

#### 2.6.2. Trolox Equivalent Antioxidant Capacity (TEAC) Assay

TEAC assay was carried out according to Bazzicalupo et al. [25] with some modifications. The radical reagent was generated in 12 h at RT and in the dark by mixing 1.7 mM ABTS with 4.3 mM K_2_S_2_O_8_. Then, the radical solution was diluted to obtain an average absorbance of 0.7 at 734 nm and used within 4 h. Ten microliters of BSE and BWE (30–240 μg/mL) were added to the reagent (200 µL) and incubated at RT for 6 min. The decrease in absorbance was recorded at 734 nm by using the same instrument and blank reported in Section 2.4.1. Trolox was used as a reference compound (1.25–10.0 μg/mL). Results were expressed as reported in Section 2.6.1.

#### 2.6.3. Ferric-Reducing Antioxidant Power (FRAP) Assay

FRAP assay was carried out according to Occhiuto et al. [20] with some modifications. Briefly, 10 µL of BSE and BWE (30–240 μg/mL) were added to 200 µL of fresh, pre-warmed (37 °C) working FRAP reagent consisting of 300 mM buffer acetate (pH 3.6), 10 mM TPTZ-40 mM HCl, and 20 mM FeCl_3_ and incubated for 4 min at RT in the dark. The absorbance was recorded at 593 nm using the same instrument and blank reported in Section 2.4.1. Trolox was used as reference compound (2.50–20.0 μg/mL). Results were expressed as reported in Section 2.6.1.

#### 2.6.4. Oxygen Radical Absorbance Capacity (ORAC) Assay

The ORAC assay was carried out according to Cornara et al. [26]. Briefly, 20 μL of BSE and BWE (3.125–25.0 μg/mL) were added to 120 μL of fresh 117 nM fluorescein and incubated for 15 min at 37 °C. Then, 60 μL of 40 mM AAPH were added to trigger the reaction, which was recorded every 30 s for 90 min (λ_ex_ 485; λ_em_ 520) by a fluorescence microplate reader (FLUOstar Omega, BMG LABTECH, Ortenberg, Germany). Trolox was used as a reference compound (0.25–2.5 μg/mL). Results were expressed as reported in Section 2.6.1.

### 2.7. Antimicrobial Activity

The following strains from the University of Messina’s *in-house* culture collection (Messina, Italy) were used for antimicrobial testing: *Staphylococcus aureus* ATCC 6538, *Escherichia coli* ATCC 10536, *Pseudomonas aeruginosa* ATCC 9027, *Candida albicans* ATCC 10231. Bacteria were grown in Mueller–Hinton broth (MHB, Oxoid, CM0405) at 37 °C (24 h), whereas the yeast cells were cultured in Sabouraud liquid medium (SLM, Oxoid, CM0147) at 30 °C (48 h). The minimum inhibitory concentration (MIC) was determined by the broth microdilution method according to CLSI M100-S22 [27] and CLSI M27-A3 [28] for bacteria and yeast, respectively.

### 2.8. Antiviral Potential

#### 2.8.1. Cells Culture and Virus

Vero cell lines (American Type Culture Collection) were propagated in minimal essential medium (EMEM) and supplemented with 6% foetal bovine serum (FBS) (Lonza, Belgium) at 37 °C under 5% CO_2_. The prototype HSV-1 (F) strain, used for the in vitro experiments, was kindly provided by Dr. Bernard Roizman (University of Chicago, Chicago, IL, USA), and the virus stock was produced and titred in Vero cells.

#### 2.8.2. Cell Proliferation Assay

The cell viability was tested by CCK-8 assay (ab228554; Abcam) according to the manufacturer’s instruction. WST-8/CCK8 tetrazolium salt is reduced by cellular dehydrogenases to an orange formazan product that is soluble in tissue culture medium. The amount of formazan produced is directly proportional to the number of living and metabolically active cells and is measured by absorbance at 460 nm. Therefore, Vero cells (2.5 × 10^4^ cells/mL) were grown in 96-well microtiter plates at 37 °C in a 5% CO_2_ incubator for 24 h. Then, they were exposed to serial dilutions of BSE (50 μg/mL, 100 μg/mL, 150 μg/mL, 200 μg/mL, and 300 μg/mL) for 72 h and incubated with CCK8 tetrazolium salt for 4 h at 37 °C in a CO_2_ incubator. The absorbance was measured at 460 nm with GloMax^®^ Multi Microplate Luminometer (Promega Corporation, 2800 Woods Hollow Road, Madison, WI, USA) and the % of cellular viability was calculated compared to untreated cells.

#### 2.8.3. Plaque Reduction Assay

The Vero cells were seeded on 24-well plates and infected with the virus inoculum for 1 h at 37 °C with gentle shaking. After the incubation time, the inoculum was removed and the monolayers were overlaid with Dulbecco’s Modified Eagle’s Medium containing 0.8% methylcellulose in the presence of the BSE at various concentrations (100 μg/mL, 150 μg/mL, 200 μg/mL). The plates were incubated at 37 °C with 5% CO_2_ for 72 h. DMSO was included as a control and used at 1% concentration. After three days, the cells were fixed, stained with crystal violet, and visualized at 10× magnification with an inverted microscope (Leica DMIL, Nuloch, Germany) for plaque detection. The data were analysed as triplicates ± standard deviation (SD) for each dilution.

### 2.9. Prebiotic Properties

A lyophilized culture of *L. acidophilus* 5 (LA5) was obtained from a commercial suspension (Solgar Italia Multinutrient S.p.A. Italy) and cultivated in de Man, Rogosa and Sharpe (MRS) broth (Oxoid, Milan, Italy) at pH 7.0, 37 °C under anaerobic condition for 24 h. The cells were collected by centrifugation (4000× *g*, 15 min, 4 °C), washed twice, re-suspended in sterile saline to achieve viable counts of approximately 6 log CFU/mL. This cell suspension (1% *v*/*v*) was individually inoculated in MRS medium (150 mL) containing BS obtained from the three different cultivars (6 Fascionello, 6 Pizzuta, and 1 Tuono) at the final concentration of 1%. The negative control was performed without addition of BS.

Immediately after the inoculum and at intervals of 24, 48, and 72 h of incubation, 100 μL from each test was serially diluted in sterile saline solution and plated on MRS agar to perform cell count by the micro drop technique [29].

The viable cells were counted after 24 h anaerobic incubation at 37 °C and the results expressed as log CFU/mL.

To evaluate the influence of BS (Fascionello and Pizzuta) on the growth and survival of the tested strain, the pH was measured and adjusted to pH 7.0 after 24 h. Immediately after the inoculum and at intervals of 2, 4, 8, 24, and 48 h of incubation, colonies were counted.

### 2.10. Statistical Analysis

Three independent experiments in triplicate (*n* = 3) were carried out for each chemical analysis and in vitro assay. The statistical significance was evaluated by one-way analysis of variance (ANOVA) followed by Student–Newman–Keuls and Tukey’s test using SigmaPlot 12.0 software (Systat Software Inc., San Jose, CA, USA). *p* < 0.05 was considered statistically significant. Moreover, agglomerative two-way hierarchical clustering analysis was carried out to highlight the nutritional and phytochemical relationships and similarities among the different cultivars investigated using the statistical JMP7 for SAS software (version 7, SAS Institute Inc., Cary, NC, USA).

## 3. Results

### 3.1. Nutritional Properties and Fatty Acids Profiles of BS Samples

The nutritional profiles of the almond BS samples belonging to the three different cultivars (Fascionello, Pizzuta, and Tuono) are reported in Table 1.

Six parameters were determined: moisture, ash, fats, proteins, fibres, and sugars. The results were expressed as g/100 g of fresh matter. Furthermore, the energy value was calculated and expressed both as Kcal and KJ.

Blanched skin appeared to be a nutritional by-product since it contained a high percentage of fibres, good protein content, and restrained quantity of fats and sugars, which also resulted in a modest value of calories. Comparing the results of the different producers (I–VII) of the Fascionello and Pizzuta cultivars, statistically significant differences (*p* < 0.05) were observed for almost all the parameters analysed, with the exception of producers II, V, and VI of the Fascionello cultivar regarding fibre, and producers III–VI, II–VI, IV, and VI of the Pizzuta cultivar regarding protein, fibre, and sugars, respectively (Table 1).

**Figure 1:**
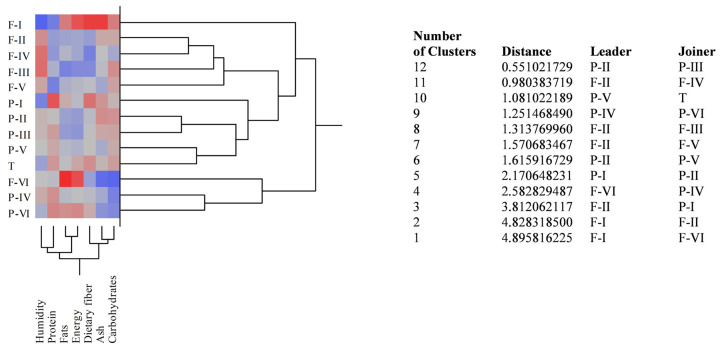
Agglomerative two-way hierarchical clustering analyses of the nutritional profiles of blanched skin (BS) samples belonging to the different producers of Fascionello (F), Pizzuta (P), and Tuono (T) cultivars.

On the contrary, no statistically significant differences in terms of nutritional parameters were detected by calculating the means and relative standard deviations of the six producers of each cultivar and comparing the three cultivars investigated. The only exception was the Fascionello cultivar, whose protein content appeared significantly (*p* < 0.05) lower than both the Pizzuta and Tuono cultivars (average values of 11.35 ± 0.31 vs. 12.23 ± 0.28 and 12.18 ± 0.10 g/100 g, respectively) (Table 1 and Figure 1).

**Figure 2:**
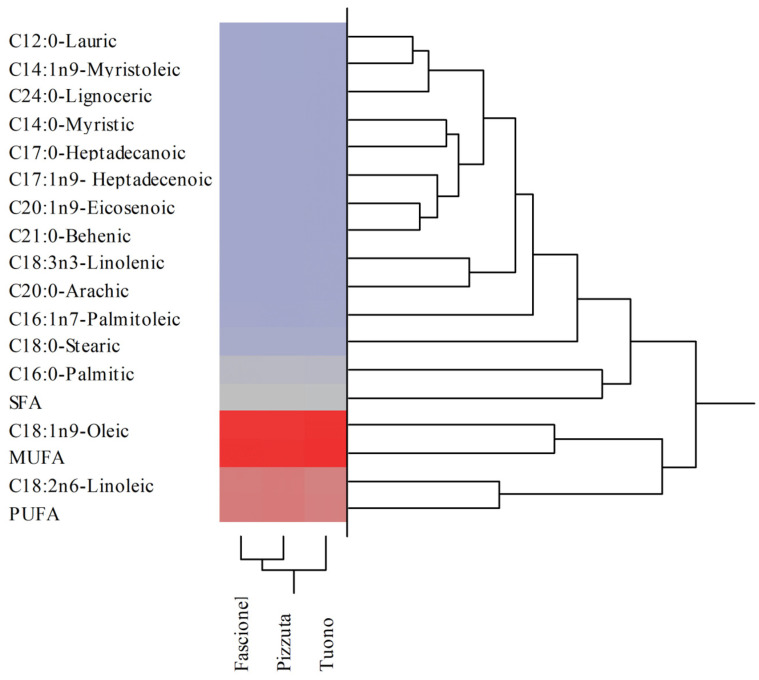
Agglomerative two-way hierarchical clustering analyses of the fatty acid profiles of blanched skin (BS) belonging to Fascionello, Pizzuta, and Tuono cultivars.

This trend of similarity between the different cultivars was even more evident when analysing fatty acid profile. Although some statistically significant values were found amongst the different cultivars, as reported in Table 2, the fatty acids profiles were superimposable, with oleic acid representing the major fatty acid, followed by linoleic, palmitic, and stearic acids. Furthermore, by grouping the fatty acids into saturated (SFA), monounsaturated (MUFA), and polyunsaturated (PUFA), the values were almost superimposable, with only the Tuono cultivar showing slightly lower values of SFA and PUFA and slightly higher MUFA (Table 2 and Figure 2).

### 3.2. Phytochemical Analyses of BSE and BWE

Preliminary screening carried out by two in vitro assays enabled quantifying the total phenols and flavonoids contents (TPC and TFC, respectively) in both BSE and BWE. The results, expressed as gallic acid and rutin equivalents, respectively (GAE and RE)/100 g of dried extract (DE), are shown in Table 3.

Given that the Fascionello and Pizzuta cultivars were provided by the same six different producers (I–VI), it was possible to compare the results obtained in terms of TPC and TFC both for BSE and BWE. The results were always statistically significantly different except for producers III and IV in TFC for BSE and producer II in TFC for BWE (Table 3).

Furthermore, statistically significant differences (*p* < 0.05) were found between the TPC and TFC values recorded in the BSE and BWE from the different producers (I–VI) for each cultivar investigated (Fascionello and Pizzuta).

However, by calculating the means and relative standard deviations (SD) of the six producers of the two different cultivars (Fascionello and Pizzuta), no statistically significant results were observed amongst the three cultivars investigated (Fascionello, Pizzuta, and Tuono), with the exception of the TPC of BWE Pizzuta compared to BWE Tuono (2.25 ± 0.88 vs. 1.00 ± 0.09 g GAE/100 g DE, *p* < 0.05) and the TFC of BWE Pizzuta and BWE Fascionello compared to BWE Tuono (0.35 ± 0.14 and 0.41 ± 0.22 vs. 0.18 ± 0.00 g RE/100 g DE, *p* < 0.05).

These preliminary data were corroborated by the results obtained by LC-DAD-ESI-MS analysis, by which the polyphenolic profile was determined quali-quantitatively. The results of the phytochemical analysis expressed as mg of each polyphenol/100 g DE are shown in Table 4. The results of the Fascionello and Pizzuta cultivars represent the mean ± SD of the six producers, which provided the BS and BW samples.

Despite the same qualitative polyphenolic profile being observed in the three different cultivars, statistically significant differences (*p* < 0.05) in terms of abundance of each polyphenol were detected (Table 4). Regarding BSE, the Pizzuta cultivar was the richest in polyphenols, followed by Fascionello and Tuono. On the contrary, regarding BWE, a higher content of polyphenols was detected in the Fascionello cultivar, followed by Tuono and Pizzuta (Table 4). BSEs were characterized by, in order of abundance, isorhamnetin-3-*O*-glucoside, catechin, kaempferol-3-*O*-glucoside, naringenin-7-*O*-glucoside, vanillic acid, epicatechin, and quercetin-3-*O*-galactoside in the Fascionello cultivar; isorhamnetin-3-*O*-glucoside, catechin, naringenin-7-*O*-glucoside, protocatechuic acid, vanillic acid, isorhamnetin, and chlorogenic acid in the Pizzuta cultivar; and isorhamnetin-3-*O*-glucoside, kaempferol-3-*O*-glucoside, quercetin-3-*O*-rhamnoside, protocatechuic acid, catechin, vanillic acid, and naringenin-7-*O*-glucoside in the Tuono cultivar (Table 4).

Conversely, BWE were characterized by, in order of abundance, isorhamnetin-3-*O*-glucoside, vanillic acid, catechin, protocatechuic acid, quercetin, and isorhamnetin-3-*O*-glucoside in the Fascionello cultivar; isorhamnetin-3-*O*-glucoside, vanillic acid, protocatechuic acid, naringenin-7-*O*-glucoside, 4-hydroxybenzoic acid, and kaempferol-3-*O*-glucoside in the Pizzuta cultivar; and isorhamnetin-3-*O*-glucoside, vanillic acid, protocatechuic acid, 4-hydroxybenzoic acid, quercetin-3-*O*-rhamnoside, and quercetin-3-*O*-galactoside in the Tuono cultivar (Table 4). The metabolites mostly distinguishing the different cultivars were catechin and epicatechin in the Fascionello cultivar, isorhamnetin, protocatechuic acid, and chlorogenic acid in the Pizzuta cultivar, and quercetin-3-*O*-rhamnoside in the Tuono cultivar. Different behaviour was instead found in the BWE, where the metabolites mostly distinguishing the cultivars were catechin, quercetin, and quercetin-3-*O*-rhamnoside for the Fascionello cultivar, kaempferol-3-*O*-glucoside and naringenin-7-*O*-glucoside for the Pizzuta cultivar, and isorhamnetin-3-*O*-glucoside and quercetin-3-*O*-galactoside for the Tuono cultivar.

**Figure 3:**
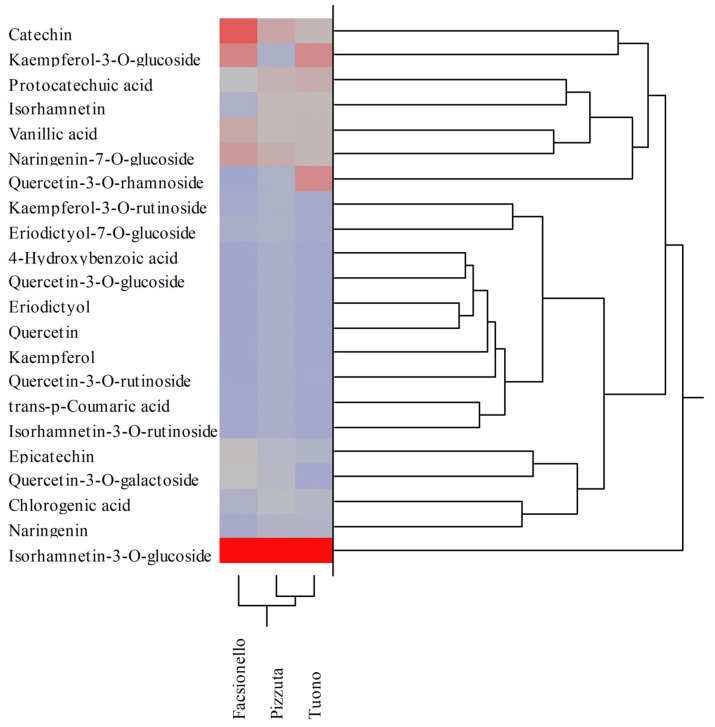
Agglomerative two-way hierarchical clustering analyses of the polyphenolic profiles of blanched skin extracts (BSE) belonging to Fascionello, Pizzuta, and Tuono cultivars.

**Figure 4:**
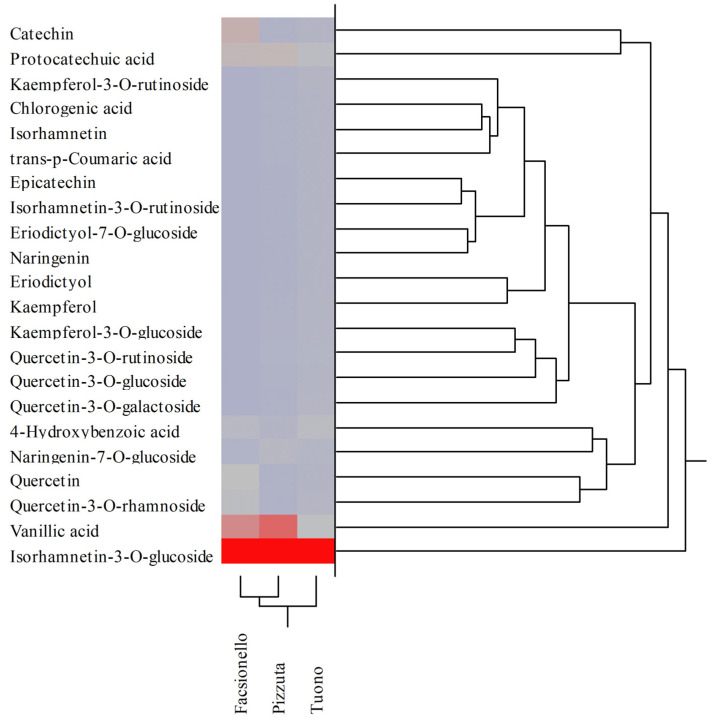
Agglomerative two-way hierarchical clustering analyses of the polyphenolic profiles of blanch water extracts (BWE) belonging to Fascionello, Pizzuta, and Tuono cultivars.

The percentage differences found in terms of expression of each metabolite appear even clearer by performing two-way clustering analyses. The colour map, indeed, allows to identify the cultivar in which the content of the specific constituent was higher (dark red to light red), lower (grey to light blue), or absent (dark blue) (Figure 3 and Figure 4 for BSE and BWE, respectively).

These multivariate analyses confirmed that the polyphenolic profiles recorded between the three different cultivars analysed were not statistically significant different. Indeed, as detected from the dendrograms shown in Figure 3 and Figure 4, the results of the agglomerative hierarchical clustering analyses, carried out on the 22 polyphenols identified and quantified in the three different cultivars (Fascionello, Pizzuta, and Tuono), showed a distance ≤1.09 both for BSE and BWE (Figure 3 and 4, respectively).

**Figure 5:**
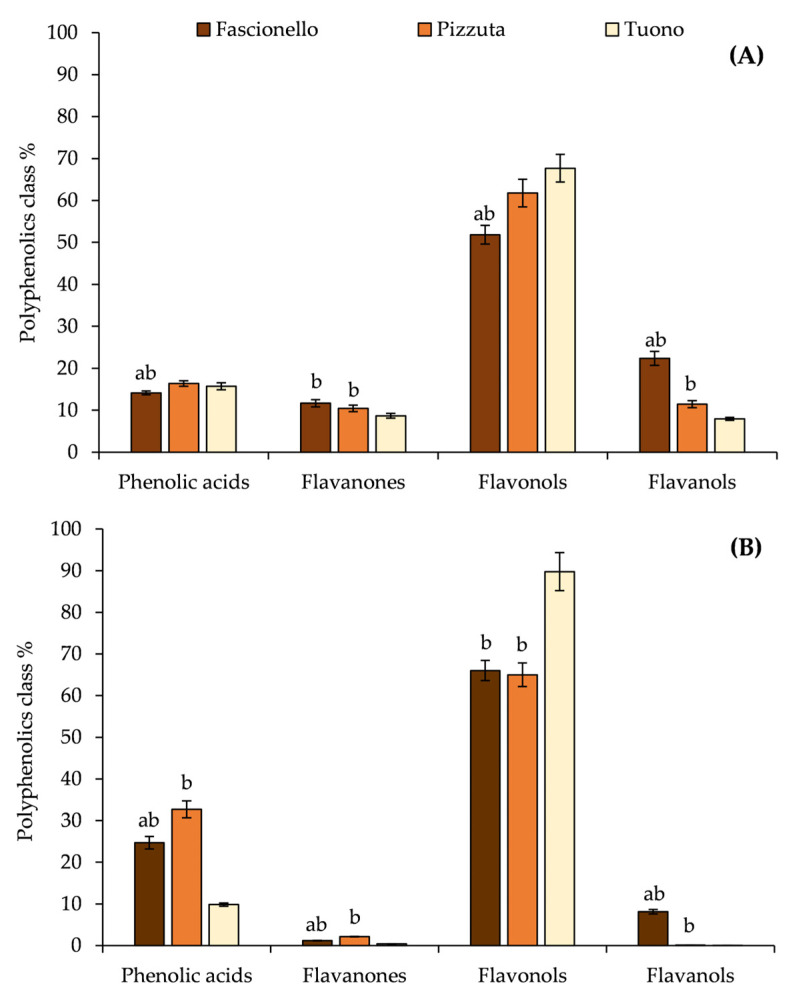
Percentage distribution (%) of the various classes of polyphenols identified in the three different cultivars investigated (Fascionello, Pizzuta, and Tuono) in blanched skin extracts (BSE, panel (**A**)) and blanch water extracts (BWE, panel (**B**)). ^a^ *p* < 0.05 vs. Pizzuta cultivar; ^b^ *p* < 0.05 vs. Tuono cultivar.

By grouping the identified and quantified polyphenols into classes, it was interesting to note, as shown in Figure 5, that there was a substantial difference between BSE and BWE in terms of the percentage distribution of the different polyphenolic classes.

Indeed, flavonols was the most abundant class of polyphenols, followed by flavanols, phenolic acids, and flavanones in BSE (Figure 5). For BWE, although flavonols remained the most abundant class, they were followed by phenolic acids, flavanones, and flavanols, except for the Fascionello cultivar, which showed a greater content of flavanols with respect to flavanones (Figure 5).

### 3.3. Antioxidant and Free-Radical Scavenging Activity of BSE and BWE

The antioxidant and free-radical scavenging properties of BSE and BWE were investigated by four in vitro assays based on different mechanisms and reaction environments (Table 5).

According to the previous results, which highlighted a higher polyphenols content for BSE with respect to BWE, whichever test was used, the activity was always higher for the BSE samples. The free-radical scavenging activity was strongly influenced by the type of molecules within the extracts, and, as such, it also varies considerably according to the test used to evaluate it. Despite this, it was interesting to note how all the analysed samples maintain the same trend, with greater activity found in the hydrogen-atoms-transfer-based tests (ORAC), followed by those with mixed behaviour (tests based on electron and hydrogen atoms transfer), such as DPPH and TEAC, and finally those based on electron transfer (FRAP).

Even in the case of antioxidant activity, although significantly different values have often been found between BSE and BWE of different cultivars from the same producer (Table 5), expressing the results as the means and relative standard deviations and comparing the three cultivars investigated, few statistically significant results (*p* < 0.05) were recorded (Table 5).

Regarding BSE, the antioxidant activity of the Fascionello cultivar appeared significantly (*p* < 0.05) higher than the Tuono cultivar only in the TEAC assay (10.01 ± 3.29 vs. 5.72 ± 0.20 g TE/100 g DE). On the contrary, the Tuono cultivar showed significantly (*p* < 0.05) higher ORAC results with respect to both the Fascionello and Pizzuta cultivars (67.69 ± 5.83 vs. 25.22 ± 5.81 and 47.76 ± 16.82 g TE/100 g DE).

On the contrary, regarding BWE, the Pizzuta cultivar showed the best results, with significantly (*p* < 0.05) higher values in all the tests carried out with respect to the Tuono cultivar, and, in the ORAC test, also with respect to the Fascionello cultivar (23.21 ± 4.68 vs. 10.96 ± 6.73 g TE/100 g DE). Conversely, the Fascionello cultivar showed significantly (*p* < 0.05) higher antioxidant activity with respect to the Tuono cultivar only in the DPPH assay (4.97 ± 3.50 vs. 1.26 ± 0.01 g TE/100 g DE).

Finally, statistically significant differences (*p* < 0.05) were recorded between the six producers of the Fascionello and Pizzuta cultivars except for producers I–V of the Pizzuta cultivar in the TEAC assay, both for BSE and BWE, and V and VI of the BSE belonging to the Pizzuta cultivar in the ORAC assay (Table 5).

### 3.4. Antimicrobial and Antiviral Activity of BSE

As no significant differences were detected amongst the three different cultivars in terms of macronutrients composition and polyphenolic profile, the antimicrobial and antiviral effects were determined using the extracts from Fascionello and Tuono cultivars. No antimicrobial effect was determined at the tested concentrations (results not shown).

#### 3.4.1. Cell Viability Assay

**Figure 6:**
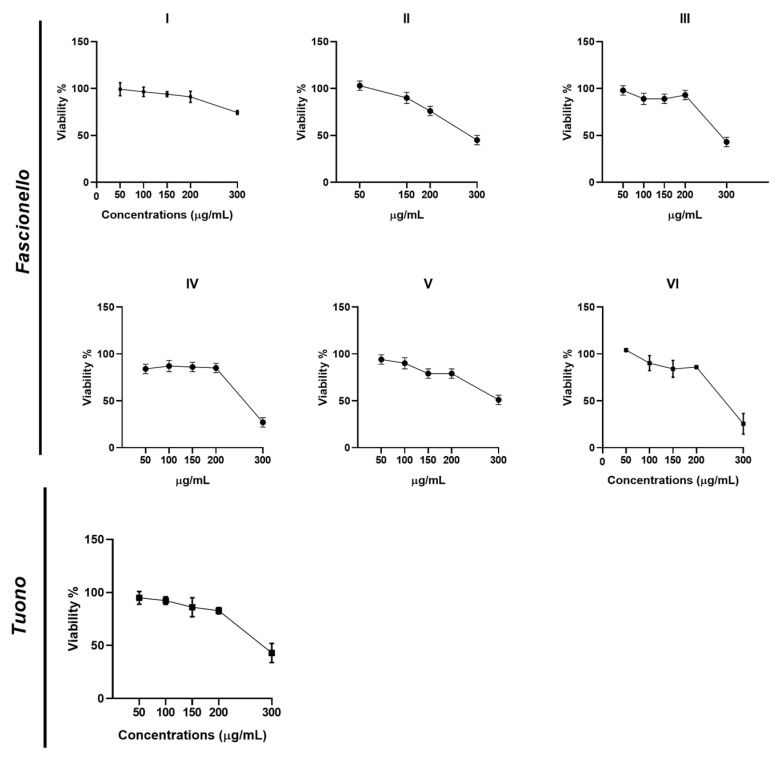
Viability assay of Vero cells treated with BSE from Fascionello and Tuono cultivars. Vero cells were treated with different concentrations of BSE (50 μg/mL, 100 μg/mL, 150 μg/mL, 200 μg/mL, and 300 μg/mL). The cells were collected 72 h post-treatment. The cell viability was carried out as described in the Materials and Methods section and graphically reported as a percentage of viability (%). The assay was performed as means of triplicates ± SD. I to VI represent different producers.

All extracts did not show a significant cytotoxic effect except at 300 µg/mL, which substantially affected cellular proliferation (Figure 6). Based on these results, the reported CC_50_ (concentration that reduced the proliferation of Vero cells by 50%) values varied between 300 and 800 μg/mL (Table 6).

#### 3.4.2. Antiviral Activity

**Figure 7:**
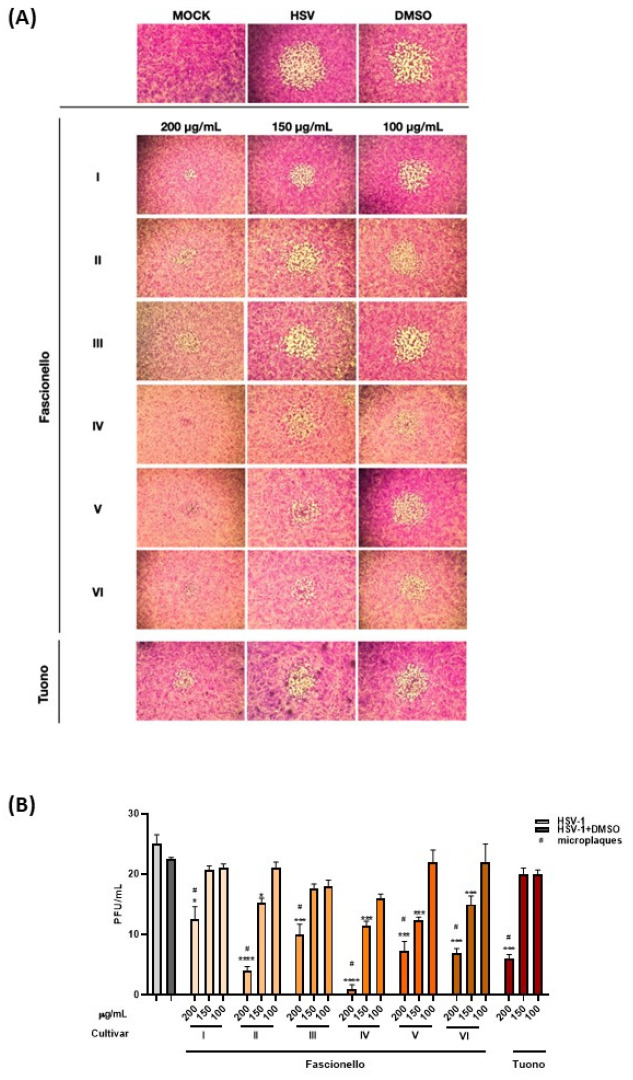
Effect of BSE on HSV-1 replication by plaque reduction assay. Vero cells were infected with HSV-1 dilutions for 1 h and then treated with BSE from Fascionello and Tuono cultivars (200 μg/mL, 150 μg/mL, and 100 μg/mL). DMSO was included as a control. Data are expressed as mean (± SD) of triplicates. # indicates the micro-plaques. * *p* < 0.05, *** *p* < 0.001, **** *p*< 0.0001 vs. HSV-1 + DMSO. I to VI represent different producers.

The in vitro susceptibility of the virus to antiviral agents was studied through plaques reduction assay, measuring the half-maximal effective concentration (EC_50_), which was calculated using non-linear regression analysis. We found that treatment with BSEs from Fascionello and Tuono cultivars caused a reduction in the number of viral plaques and a change in morphology compared to the untreated infected cells. A dose-dependent antiviral activity for all BSEs was detected, exhibiting a significant inhibitory effect at 200 μg/mL. Plaque morphological changes (Figure 7, panels A,B) were displayed in treated samples compared to HSV-1 plaque morphology. The EC_50_ values, CC_50_, and SI are shown in Table 6.

Comparing the cytotoxicity profile to the antiviral effect, we obtained the SI value, which indicates the selectivity of the extracts towards the virus. Higher SI values indicate that the BSEs are considered sufficiently active. Our data report high SI values for BSE I of the Fascionello cultivar and an SI > 1 for the other BSE, indicating considerable efficacy.

### 3.5. Prebiotic Effects of BS Samples

The viable cell counts (log CFU/mL) of LA5 in a medium containing the BS samples belonging to the different tested cultivars (I–VI for Fascionello and Pizzuta and one for Tuono) and control during fermentation are shown in Table 7.

At the beginning of cultivation, all tested media showed 6.23 logs CFU/mL. On the other hand, during cultivation, the viable cell counts, especially in some media containing BS, showed a lower decrease when compared to the control. The assay was repeated by correcting the pH to 7.0 after 24 h in order to assess the effect of pH on the growth of the microorganism. However, no significant results have been found with respect to the previous scenario, proving that pH variation cannot be the cause of that variability. The counts of LA5 every 24 h for a total fermentation time of 72 h were reported in Table 7. The viable cell counts after 24 h reached, in most cases, significantly (*p* < 0.05) greater results than the control. These results continue to remain significantly higher than the control even at 48 h. On the contrary, at 72 h, statistically significant results were observed only for producers IV and V of the Fascionello cultivar, for producers I–III of the Pizzuta cultivar, and for the Tuono cultivar.

No statistically significant results were observed between the six producers of the Fascionello and Pizzuta cultivars at 24 h (Table 7). On the contrary, statistically significant differences (*p* < 0.05) were recorded at 48 h and 72 h for the Fascionello cultivar except for producers V and VI and only at 72 h for the Pizzuta cultivar except for producers V and VI (Table 7).

## 4. Discussion

Over the past 15 years, almond by-products, legally recognized as special waste [9], have been investigated from different points of view, aiming at recovery from a circular economy perspective. Amongst the different applications proposed, such as livestock feeding [30], renewable energy production [31], and use as active carbon absorbents [32], a nutraceutical perspective for BS and BW, exploring their suitability as sources of functional ingredients to be applied in the food, pharmaceutical, and cosmetics fields, has been recently adopted.

Particular attention was paid to the polyphenolic content of these by-products given that the almond skin contains 60–80% of the whole seed polyphenols [33]. These secondary metabolites are primarily responsible for the numerous health properties attributed to almond skins and blanch water [5,8,13,14,15,34,35,36,37,38,39,40].

The percentage of polyphenols released from the skins into the BW generally ranged from 74–88% [41]. Previous studies showed that total phenols content may differ from season to season based on weather differences and agricultural practices [38,42] as well as ripeness, processing, and storage [16].

However, Hughey and co-workers [41] demonstrated that the rate by which polyphenols leached from the skins into the BW follows a first order kinetic and is strictly related to time and temperature of the blanching process adopted. Furthermore, the sometimes-fluctuating behaviour in terms of polyphenolic profile found in BS and BW also depends on the physico-chemical features of each polyphenol. The authors have, in fact, verified that some non-polar compounds, such as kaempferol and isorhamnetin, precipitate due to their poor solubility in water, just as other more soluble compounds can precipitate due to saturation processes [41]. A previous study has also highlighted high fermentative power of BW, probably due to the high concentration of glycosylated flavonoids [9]. This is certainly a very important aspect to consider in using this by-product as a source of polyphenols or to treat it adequately as raw material for food, pharmaceutical, and cosmetic use. To date, this is the first study comparing two industrial Sicilian by-products from the same cultivar and with the same origin, in turn exploring three different cultivars. This aspect is of fundamental importance to understand whether any variations depend specifically on the cultivar investigated.

The analyses carried out over the years on almond skins from different origins and harvests suggest that they can be considered an excellent source of polyphenols (≥10 mg/g) [10]. Despite several factors, including the extraction method, possibly affecting meaningful comparison, our results of total phenols content were comparable to those previously obtained by our group and other researchers [5,15,35,42]. Moreover, according to previous results [5,15], BS showed the best antioxidant activity, followed by BW.

The polyphenolic profile of almond skin was characterized by the prevalence of flavanols and flavonols, followed by phenolic acids and flavanones [43]. However, depending on the almond origin, and, therefore, on the pedo-climatic characteristics, type of cultivation, cultivar, and seasonality, the polyphenolic profile can also significantly change between the different natural skins and consequently also in processing by-products. Furthermore, the blanching and drying steps may favour degradation, hydrolysis, and decomposition processes [44,45,46,47]. However, skin blanching would favour solubilization of the more hydrophilic polyphenols, such as flavonoid glycosides, flavan-3-ol monomers, quercetin against kaempferol, naringenin against eriodyctiol, and so on. These combined events could explain the differences observed in phenolic profiles of almond skins after the different industrial processes. When comparing the polyphenolic profiles of Californian [17] and Sicilian [5] almond skins, the first was characterized by a clear prevalence of flavonols and flavanols (39.55% and 31.02%, respectively), followed by flavanones (15.21%) and phenolic acids (14.22%), whereas the latter was characterized by a clear prevalence of flavanones and flavonols (50.51% and 46.95%, respectively), followed by phenolic acids (2.03%) and flavanols (0.51%). More interestingly, the expression of individual metabolites also changed conspicuously. Catechin was the predominant flavonoid, followed by naringenin-7-*O*-glucoside (absent in Sicilian), quercetin, epicatechin, isorhamnetin-3-*O*-rutinoside, kaempferol-3-*O*-rutinoside, kaempferol, and isorhamnetin-3-*O*-glucoside in Californian skins [17]. On the contrary, the most abundant metabolites were naringenin, kaempferol-3-*O*-glucoside, kaempferol, quercetin-3-*O*-rutinoside, quercetin, isorhamnetin-3-*O*-glucoside, and vanillic acid in Sicilian skins [5]. Other studies reported isorhamnetin-3-*O*-rutinoside as the main polyphenol [37,47]. The antioxidant activity of these polyphenols was widely investigated [48], with flavan-3-ols, flavonols, and flavanones proving to be the compounds with the greatest influence on the total antioxidant capacity [42]. The differences in amount and qualitative profile of phytochemicals may also explain the lack of antimicrobial effect detected in the present study compared with previous research [5].

Furthermore, the not negligible aspect is that, often, in previous studies, the cultivar to which the waste products refer to, is not reported [8,14,15,17,35,42,48,49]. Only in very few cases, the authors made a comparison between waste products and natural skin (NS), which is of fundamental importance for understanding the real loss of polyphenols and their distribution in the two by-products, BS and BW. Our previous study carried out on Sicilian NS, BS, and BW of a Pizzuta cultivar provided by Avola Almond Consortium showed that the polyphenolic profile of NS remains unchanged after the blanching process [5]. Polyphenols remain in part in the BS and are partly released into the BW, with the total content of the two by-products accounting for 49.74% with respect to the NS total polyphenols content [5]. It is likely that other polyphenols may precipitate due to the highlighted above processes of saturation and poor solubility in the BW. However, the very important aspect emerging from this study is that the blanching process adopted is not able to modify at least the parent polyphenols profile of the Sicilian almond. However, the loss in terms of active compounds is important and comparable with that of other nuts [15,44] and, therefore, worthy of being improved from a technological point of view.

Beyond polyphenols, other nutrients, including fats, carbohydrates, proteins, and fibres, certainly play a pivotal role for BS recovery and upgrade. The protein and sugars contents reported in the present work were consistent with our previous investigation [15]. However, this study showed lower fat content (on average 11.64% vs. 24.2%) and higher dietary insoluble fibre content (on average 55.43% vs. 45.10%) compared with Mandalari et al. [15]. Regarding fatty acids, a superimposable profile was found with MUFA, which represented the main class (56.0%), followed by PUFA and SFA. Based on these data, a further advantage of BS recovery is the low sugars and fat contents and the high dietary fibre content with respect to potential benefits on gut health.

We have previously evaluated the prebiotic properties of Californian almond BS by using a full model of the gastrointestinal tract followed by colonic fermentation using mixed faecal bacterial cultures [14]. BS significantly increased the population of *Bifidobacteria* and *Clostridium coccoides/Eubacterium rectale* group, resulting in a prebiotic index well comparable with that of commercial prebiotic fructo-oligosaccharides (FOS). These preliminary data were corroborated by an in vivo study in which the authors investigated the prebiotic effects of almond skin intake on 48 healthy adult volunteers in comparison with roasted almonds and FOS for 6 weeks [50]. Significant increases in populations of *Bifidobacterium* spp. and *Lactobacillus* spp. were observed in faecal samples of subjects supplemented with almond or almond skin, showing interesting prebiotic effects. Although our results in terms of prebiotic potential are still preliminary, as tested on a single *Lactobacillus* strain, they confirmed what was previously observed, making BS worthy of further investigation in relation to its prebiotic effect. Any food that reaches the colon without being digested is a prebiotic candidate. From this point of view, BS, which contains approximately 50% dietary fibre, is an ideal candidate. Moreover, BS contains a significant amount of polyphenols, some of which are often slowly absorbed or partially unabsorbed, thus reaching the gut lumen [50], where they could affect the microbiota, conferring positive gut health benefits [51]. Accordingly, it seems that fibres and polyphenols cooperate in conferring prebiotic effects on BS. As a result, the activities of faecal bacterial enzymes also change. In particular, the activity of β-galactosidase increases, and the activities of β-glucuronidase, nitroreductase, and azoreductase decrease. This is of particular importance because impairment in glycosidase activity, mainly synthesized by *Bifidobacteria* and *Lactobacilli*, may induce failure in metabolism of unabsorbed carbohydrates, such as reported in inflammatory bowel diseases [52].

Our in vitro study on cell cultures has reported that BSE from Fascionello and Tuono did not exhibit cytotoxic effects except at high concentrations and displayed significant antiviral effects against HSV-1, probably ascribable to the high concentration of flavonols, mainly isorhamnetin glycosides, flavanols, such as catechin and epicatechin, and flavanones, such as naringenin-7-*O*-glucoside, as previously observed for other almond NS and BS extracts [17]. This virus represents the most prevalent human virus worldwide and commonly causes recurrent infections, ranging from localized ulcers to severe disseminated infections, especially in immunocompromised patients. The viral infection produces marked cytopathogenic effect with lysis of the host cells and dissemination of the virus to neighbouring cells. Extracts rich in polyphenols are known to directly interfere with viral particles through inhibition of the virus attachment and exhibit a specific antiherpetic property [17,18,53]. Moreover, comparing the cytotoxicity profile to the antiviral effect, we obtained high SI values, indicating considerable antiviral efficacy.

## 5. Conclusions

The present study is the first comparing the polyphenolic and nutritional profile and antioxidant, antimicrobial, antiviral, and prebiotic effects of BS and BW from different almond cultivars grown within the same production area in Sicily. We have demonstrated that the cultivar was not the discriminating factor regarding the chemical and biological properties of BS and BW. Conversely, it is possible that the pedo-climatic conditions of cultivation sites and the industrial peeling process play a key role in the nutritional and polyphenolic profile and biological properties of these by-products. These variables are reflected in the composition of the originated by-products and, therefore, affect their potential use in the pharmaceutical, food, and nutraceutical sectors.

We believe that integrated work amongst researchers and manufacturing companies becomes of primary importance as only careful sampling of waste matrices can lead to more truthful, reproducible, and comparable results, even between different almond cultivars and across different production years.

This could enable reconsidering timesaving, green, and efficient processes for sectorial industries, reducing environmental impact and providing added value to by-products for their more profitable reuse.

## Figures and Tables

**Table 1:** Characterization of the nutritional profiles of the blanched skin samples belonging to three different almonds’ cultivars: Fascionello (I–VI), Pizzuta (I–VI), and Tuono. The results, which represent the average ± standard deviation of three independent experiments in triplicate (*n* = 3), were expressed as g/100 g of fresh weight, whereas energy values were expressed both in Kcal and KJ. Numbers from I to VI represent different producers.

Cultivar	Moisture	Ash	Fats	Proteins	Fibres	Sugars	Energy	
	g/100 g						Kcal	KJ
Fascionello								
I	6.43 ± 0.12 ^aA^	3.41 ± 0.06 ^aA^	13.37 ± 0.67 ^aA^	10.99 ± 0.42 ^aD^	60.25 ± 1.22 ^A^	5.55 ± 0.08 ^aJ^	307 ± 6.52 ^aL^	1258 ± 12.44 ^aL^
II	16.55 ± 0.85 ^aB^	2.64 ± 0.02 ^aF^	10.63 ± 0.28 ^G^	11.37 ± 0.21	53.65 ± 1.27	5.16 ± 0.12 ^B^	269 ± 2.88 ^M^	1104 ± 10.67 ^G^
III	18.39 ± 0.34 ^aC^	2.42 ± 0.03 ^aC^	9.5 ± 0.12 ^aE^	11.61 ± 0.19 ^I^	52.67 ± 1.28 ^aI^	5.41 ± 0.06 ^aK^	259 ± 1.95 ^E^	1062 ± 8.67 ^E^
IV	18.22 ± 0.58 ^aC^	2.46 ± 0.02 ^aC^	11.22 ± 0.41 ^D^	11.31 ± 0.26 ^a^	52.15 ± 1.35 ^aI^	4.64 ± 0.04 ^aC^	269 ± 2.43 ^aC^	1103 ± 12.66 ^aD^
V	15.24 ± 0.67 ^aD^	2.21 ± 0.03 ^D^	11.07 ± 0.23 ^D^	11.03 ± 0.22 ^aD^	55.23 ± 1.17	5.22 ± 0.14 ^D^	275 ± 2.66 ^D^	1128 ± 14.62 ^D^
VI	13.67 ± 0.24 ^a^	1.63 ± 0.01 ^a^	15.35 ± 0.37 ^a^	11.76 ± 0.18	53.77 ± 2.05	3.82 ± 0.03 ^a^	308 ± 3.54 ^a^	1263 ± 12.08 ^a^
Pizzuta								
I	8.88 ± 0.06 ^A^	2.78 ± 0.04 ^E^	12.17 ± 0.42 ^H^	12.78 ± 0.25 ^J^	58.34 ± 1.42 ^H^	5.05 ± 0.22 ^K^	276 ± 2.67 ^N^	1132 ± 13.65 ^N^
II	14.33 ± 0.22 ^E^	2.84 ± 0.05 ^F^	10.55 ± 0.25 ^E^	11.85 ± 0.27 ^D^	55.07 ± 1.08	5.36 ± 0.14 ^K^	265 ± 2.38 ^E^	1087 ± 7.56 ^E^
III	14.28 ± 0.12 ^E^	2.65 ± 0.04 ^E^	10.35 ± 0.46 ^E^	12.14 ± 0.32	55.37 ± 1.32	5.21 ± 0.07 ^K^	263 ± 4.42 ^E^	1078 ± 6.88 ^E^
IV	14.93 ± 0.08 ^C^	2.24 ± 0.07 ^C^	11.37 ± 0.38 ^D^	12.24 ± 0.11	55.08 ± 1.88	4.14 ± 0.04	278 ± 3.62 ^D^	1140 ± 10.05 ^D^
V	13.69 ± 0.04 ^D^	2.26 ± 0.04 ^D^	11.35 ± 0.18 ^D^	12.04 ± 0.38	55.53 ± 1.57	5.13 ± 0.18 ^K^	273 ± 4.08 ^D^	1120 ± 12.22 ^D^
VI	12.27 ± 0.33	2.01 ± 0.08	12.88 ± 0.24	12.35 ± 0.08	56.25 ± 1.62	4.24 ± 0.21	293 ± 2.44	1201 ± 8.48
Tuono	11.16 ± 0.21	2.55 ± 0.06	11.56 ± 0.36	12.18 ± 0.10	57.28 ± 1.29	5.27 ± 0.08	285 ± 1.82	1169 ± 6.54

^a^ *p* < 0.05 vs. Pizzuta cultivar from the same producers; ^A^ *p* < 0.05 vs. producers II–VI of the same cultivar; ^B^ *p* < 0.05 vs. producers III, IV, and VI of the same cultivar; ^C^ *p* < 0.05 vs. producers V and VI of the same cultivar; ^D^ *p* < 0.05 vs. producer VI of the same cultivar; ^E^ *p* < 0.05 vs. producers IV–VI of the same cultivar; ^F^ *p* < 0.05 vs. producers III–VI; ^G^ *p* < 0.05 vs. producers III and VI of the same cultivar; ^H^ *p* < 0.05 vs. producers II and III of the same cultivar; ^I^ *p* < 0.05 vs. producer V of the same cultivar; ^J^ *p* < 0.05 vs. producers II and IV–VI of the same cultivar; ^K^ *p* < 0.05 vs. producers IV and VI of the same cultivar; ^L^ *p* < 0.05 vs. producers II–V of the same cultivar; ^M^ *p* < 0.05 vs. producers III, V, and VI of the same cultivar; ^N^ *p* < 0.05 vs. producers II, III, and VI of the same cultivar.

**Table 2:** Characterization of the fatty acids profiles by GC–FID and GC–MS analyses of blanched skin samples belonging to three different almonds’ cultivars: Fascionello, Pizzuta, and Tuono. For the Fascionello and Pizzuta cultivars, the average data of the six producers were reported. The results, expressed as percentage (%) of the total fatty acids identified and quantified, represent the mean ± standard deviation of three independent experiments in triplicate (*n* = 3).

Fatty Acids	Fascionello	Pizzuta	Tuono
C12:0-Lauric	0.06 ± 0.00 ^a^	0.04 ± 0.00	0.06 ± 0.00
C14:0-Myristic	0.09 ± 0.00 ^b^	0.10 ± 0.01	0.03 ± 0.00
C14:1n9-Myristoleic	0.05 ± 0.00	0.05 ± 0.00	0.05 ± 0.00
C16:0-Palmitic	8.38 ± 0.18	8.33 ± 0.42	7.92 ± 0.24
C16:1n7-Palmitoleic	0.77 ± 0.02 ^b^	0.81 ± 0.03 ^b^	0.68 ± 0.03
C17:0-Heptadecanoic	0.11 ± 0.00 ^ab^	0.15 ± 0.01 ^b^	0.07 ± 0.00
C17:1n9- Heptadecenoic	0.10 ± 0.00 ^a^	0.13 ± 0.01	0.12 ± 0.01
C18:0-Stearic	2.38 ± 0.12	2.39 ± 0.12	2.18 ± 0.12
C18:1n9-Oleic	54.79 ± 1.24	54.14 ± 2.78	56.69 ± 3.08
C18:2n6-Linoleic	32.56 ± 2.08	33.05 ± 1.49	31.36 ± 1.52
C18:3n3-Linolenic	0.31 ± 0.02	0.33 ± 0.02 ^b^	0.27 ± 0.02
C20:0-Arachic	0.22 ± 0.01	0.24 ± 0.01	0.25 ± 0.01
C20:1n9-Eicosenoic	0.07 ± 0.00 ^ab^	0.11 ± 0.00	0.12 ± 0.01
C21:0-Behenic	0.08 ± 0.00 ^b^	0.09 ± 0.00 ^b^	0.12 ± 0.01
C24:0-Lignoceric	0.06 ± 0.00 ^b^	0.07 ± 0.00	0.08 ± 0.00
SFA	11.36	11.39	10.71
MUFA	55.77	55.24	57.66
PUFA	32.87	33.38	31.63

SFA, saturated fatty acids; MUFA, monounsaturated fatty acids; PUFA, polyunsaturated fatty acids; ^a^ *p* < 0.05 vs. Pizzuta cultivar; ^b^ *p* < 0.05 vs. Tuono cultivar.

**Table 3:** Total phenols and flavonoids contents (TPC and TFC, respectively) quantified by in vitro colorimetric assays. Results, which represent the average ± standard deviation of three independent experiments in triplicate (*n* = 3), were expressed as gallic acid and rutin equivalents (GAE and RE, respectively)/100 g of dried extract (DE). Numbers from I to VI represent different producers.

Cultivar	Blanched Skin		Blanch Water	
	TPC (g GAE/100 g DE)	TFC (g RE/100 g DE)	TPC (g GAE/100 g DE)	TFC (g RE/100 g DE)
Fascionello				
I	5.07 ± 0.36 ^aA^	1.20 ± 0.03 ^aN^	2.08 ± 0.68 ^aG^	0.77 ± 0.03 ^aA^
II	3.97 ± 0.09 ^aE^	0.95 ± 0.01 ^aO^	2.20 ± 0.13 ^aO^	0.40 ± 0.01 ^O^
III	3.88 ± 0.29 ^aE^	0.84 ± 0.01 ^K^	1.18 ± 0.03 ^aK^	0.23 ± 0.01 ^aQ^
IV	2.93 ± 0.02 ^aC^	0.89 ± 0.02 ^D^	1.69 ± 0.08 ^aC^	0.56 ± 0.03 ^aC^
V	2.48 ± 0.20 ^aD^	0.86 ± 0.02 ^aD^	1.30 ± 0.10 ^aD^	0.29 ± 0.01 ^aD^
VI	2.24 ± 0.07 ^a^	0.70 ± 0.01 ^a^	0.56 ± 0.01 ^a^	0.22 ± 0.02 ^a^
Pizzuta				
I	4.39 ± 0.18 ^A^	0.88 ± 0.03 ^P^	3.36 ± 0.04 ^O^	0.60 ± 0.01 ^A^
II	3.65 ± 0.13 ^O^	0.78 ± 0.03 ^D^	3.26 ± 0.10 ^O^	0.35 ± 0.03 ^Q^
III	7.07 ± 0.05 ^E^	0.81 ± 0.04 ^C^	2.28 ± 0.08 ^E^	0.37 ± 0.01 ^E^
IV	3.85 ± 0.01 ^C^	0.88 ± 0.07 ^C^	1.37 ± 0.05 ^C^	0.23 ± 0.02 ^D^
V	3.25 ± 0.09 ^D^	0.70 ± 0.06 ^D^	1.70 ± 0.06 ^D^	0.20 ± 0.01 ^D^
VI	1.72 ± 0.04	0.52 ± 0.02	1.55 ± 0.06	0.31 ± 0.01
Tuono	2.76 ± 0.08	0.91 ± 0.01	1.00 ± 0.09	0.18 ± 0.00

^a^ *p* < 0.05 vs. Pizzuta cultivar from the same producers. ^A^ *p* < 0.05 vs. producers II–VI of the same cultivar; ^C^ *p* < 0.05 vs. producers V and VI of the same cultivar; ^D^ *p* < 0.05 vs. producer VI of the same cultivar; ^E^ *p* < 0.05 vs. producers IV–VI of the same cultivar; ^G^ *p* < 0.05 vs. producers III and VI of the same cultivar; ^K^ *p* < 0.05 vs. producers IV and VI of the same cultivar; ^N^ *p* < 0.05 vs. producers II, III, and VI of the same cultivar; ^O^ *p* < 0.05 vs. producers III–VI of the same cultivar; ^P^ *p* < 0.05 vs. producers II, V, and VI of the same cultivar; ^Q^ *p* < 0.05 vs. producers IV and V of the same cultivar.

**Table 4:** Qualitative and quantitative determination of polyphenols in blanched skin and blanch water samples belonging to three almonds’ cultivars (Fascionello, Pizzuta, and Tuono) by LC-DAD-ESI-MS analysis. For the Fascionello and Pizzuta cultivars, the average data of the six producers were reported. Results, which represent the average ± standard deviation of three independent experiments in triplicate (*n* = 3), were expressed as mg of each polyphenol/100 g of dried extract (DE).

Polyphenols	RT (min)	λ_max_ (nm)	[M-H]^−^	Blanched Skin			Blanch Water		
				Fascionello	Pizzuta	Tuono	Fascionello	Pizzuta	Tuono
*Hydroxybenzoic acids*									
Protocatechuic acid	7.04	258;293	137	9.37 ± 0.12 ^ab^	16.69 ± 0.72 ^b^	11.98 ± 0.58	3.03 ± 0.17 ^b^	2.91 ± 0.10 ^b^	1.38 ± 0.08
4-Hydroxybenzoic acid	12.00	253	153	0.04 ± 0.00 ^ab^	0.09 ± 0.00 ^b^	0.01 ± 0.00	1.23 ± 0.03 ^b^	0.44 ± 0.02 ^b^	1.31 ± 0.06
Vanillic acid	16.00	262;291	167	16.50 ± 0.28 ^ab^	13.46 ± 0.65 ^b^	9.58 ± 0.48	7.70 ± 0.31 ^ab^	11.67 ± 0.72 ^b^	1.98 ± 0.05
*Hydroxycinnamic acids*									
Chlorogenic acid	20.50	291;319	353	4.38 ± 0.08 ^a^	8.66 ± 0.44 ^b^	4.53 ± 0.21	0.03 ± 0.00 ^ab^	0.03 ± 0.00 ^b^	0.01 ± 0.00
trans-*p*-Cumaric acid	22.80	309	163	0.55 ± 0.01 ^ab^	n.d.	0.01 ± 0.00	n.d.	0.07 ± 0.00	n.d.
*Flavanones*									
Eriodictyol-7-*O*-glucoside	29.71	283	449	3.20 ± 0.10 ^ab^	2.03 ± 0.05 ^b^	1.08 ± 0.02	0.01 ± 0.00	0.01 ± 0.00	0.01 ± 0.00
Naringenin-7-*O*-glucoside	32.43	282	433	20.59 ± 0.34 ^ab^	18.88 ± 0.67 ^b^	9.41 ± 0.23	0.46 ± 0.02 ^ab^	0.97 ± 0.03 ^b^	0.11 ± 0.01
Eriodictyol	35.66	287	287	0.01 ± 0.00	0.01 ± 0.00	n.d.	0.09 ± 0.00 ^ab^	0.01 ± 0.00	0.02 ± 0.00
Naringenin	40.26	289	271	1.66 ± 0.02 ^ab^	3.91 ± 0.17	3.94 ± 0.11	0.01 ± 0.00 ^b^	0.01 ± 0.00 ^b^	0.04 ± 0.00
*Flavonols*									
Quercetin-3-*O*-galactoside	32.36	253;354	463	10.10 ± 0.35 ^ab^	6.98 ± 0.24 ^b^	0.07 ± 0.00	0.02 ± 0.00 ^ab^	0.07 ± 0.00 ^b^	0.28 ± 0.02
Quercetin-3-*O*-rutinoside	32.41	254;354	609	0.11 ± 0.01 ^a^	0.04 ± 0.00 ^b^	0.12 ± 0.00	0.05 ± 0.00 ^ab^	0.19 ± 0.01 ^b^	0.13 ± 0.01
Quercetin-3-*O*-glucoside	32.64	254;354	463	0.02 ± 0.00 ^a^	0.06 ± 0.00 ^b^	0.02 ± 0.00	0.06 ± 0.00 ^a^	0.13 ± 0.01 ^b^	0.07 ± 0.00
Kaempferol-3-*O*-rutinoside	33.96	265;348	593	1.50 ± 0.02 ^ab^	1.18 ± 0.03 ^b^	0.40 ± 0.02	0.02 ± 0.00 ^ab^	0.07 ± 0.00	0.07 ± 0.00
Kaempferol-3-*O*-glucoside	34.34	264;347	447	26.58 ± 0.37 ^ab^	1.00 ± 0.02 ^b^	20.13 ± 0.74	0.03 ± 0.00 ^ab^	0.26 ± 0.02 ^b^	0.06 ± 0.00
Quercetin-3-*O*-rhamnoside	34.36	257;358	447	n.d.	1.22 ± 0.02 ^b^	19.63 ± 0.86	1.82 ± 0.02 ^ab^	0.12 ± 0.01 ^b^	0.31 ± 0.02
Isorhamnetin-3-*O*-rutinoside	34.63	254;354	624	0.44 ± 0.01	n.d.	n.d.	n.d.	n.d.	n.d.
Isorhamnetin-3-*O*-glucoside	34.88	254;353	477	70.60 ± 1.88 ^ab^	122.94 ± 4.67 ^b^	63.07 ± 2.36	27.91 ± 1.06 ^b^	29.09 ± 0.55 ^b^	41.71 ± 2.65
Quercetin	39.39	255;370	301	0.01 ± 0.00	0.01 ± 0.00	n.d.	2.10 ± 0.03 ^ab^	0.02 ± 0.00	0.01 ± 0.00
Kaempferol	43.66	264;365	285	0.11 ± 0.00	n.d.	n.d.	0.08 ± 0.00 ^a^	0.05 ± 0.00 ^b^	0.07 ± 0.00
Isorhamnetin	44.47	253;368	315	3.70 ± 0.18 ^ab^	13.44 ± 0.56 ^b^	9.14 ± 0.43	0.01 ± 0.00 ^ab^	0.04 ± 0.00	0.04 ± 0.00
*Flavanols*									
Catechin	18.65	279	289	38.26 ± 1.24 ^ab^	21.73 ± 0.88 ^b^	9.69 ± 0.27	3.95 ± 0.06 ^ab^	0.05 ± 0.00 ^b^	0.01 ± 0.00
Epicatechin	23.64	279	289	10.60 ± 0.27 ^ab^	5.46 ± 0.14 ^b^	3.53 ± 0.12	n.d.	0.01 ± 0.00	n.d.
Total polyphenols (mg/100 g DE)				218.35	237.80	166.33	48.62	46.21	47.63

^a^ *p* < 0.05 vs. Pizzuta cultivar; ^b^ *p* < 0.05 vs. Tuono cultivar. Italics indicate the different classes of polyphenols identified.

**Table 5:** Antioxidant and free-radical scavenging activity determined by spectrophotometric and spectrofluorimetric tests based on different environments and reaction mechanisms. Results, which represent the average ± standard deviation of three independent experiments in triplicate (*n* = 3), were expressed as g of trolox equivalents (TE)/100 g of dried extract (DE). Numbers from I to VI represent different producers.

Cultivar	Blanched Skin				Blanching Water			
	DPPH	FRAP	TEAC	ORAC	DPPH	FRAP	TEAC	ORAC
	g TE/100 g DE				g TE/100 g DE			
Fascionello								
I	11.79 ± 0.16 ^aA^	5.43 ± 0.30 ^aJ^	15.25 ± 1.11 ^aA^	34.21 ± 1.48 ^aA^	7.44 ± 0.68 ^aR^	3.32 ± 0.07 ^aJ^	5.57 ± 0.62 ^aS^	20.81 ± 0.14 ^aA^
II	10.43 ± 0.25 ^aM^	3.93 ± 0.22 ^O^	8.45 ± 0.19 ^G^	28.23 ± 0.69 ^aJ^	4.33 ± 0.12 ^aM^	2.38 ± 0.11 ^aO^	2.23 ± 0.10 ^aG^	10.48 ± 0.09 ^aE^
III	8.92 ± 0.59 ^aE^	5.12 ± 0.50 ^aE^	12.81 ± 1.09 ^aJ^	27.22 ± 1.15 ^aJ^	2.65 ± 0.18 ^aJ^	0.89 ± 0.03 ^aE^	1.49 ± 0.01 ^aE^	3.87 ± 0.13 ^aE^
IV	10.55 ± 0.08 ^aC^	6.01 ± 0.26 ^aC^	8.93 ± 0.70 ^D^	22.85 ± 1.40 ^aC^	7.92 ± 0.36 ^aC^	3.09 ± 0.16 ^C^	6.63 ± 0.27 ^aD^	10.54 ± 0.50 ^aC^
V	7.61 ± 0.17 ^aD^	3.31 ± 0.32 ^aD^	7.99 ± 0.52 ^D^	20.34 ± 0.77 ^aD^	3.99 ± 0.23 ^aD^	3.08 ± 0.42 ^D^	6.05 ± 0.46 ^aD^	16.22 ± 0.32 ^aD^
VI	3.84 ± 0.02 ^a^	2.61 ± 0.16 ^a^	6.65 ± 0.53 ^a^	18.50 ± 1.02 ^a^	0.83 ± 0.01 ^a^	0.92 ± 0.03 ^a^	1.34 ± 0.13 ^a^	3.83 ± 0.08 ^a^
Pizzuta								
I	7.48 ± 0.15 ^O^	6.49 ± 0.57 ^A^	8.38 ± 0.54 ^D^	48.16 ± 2.46 ^O^	5.30 ± 0.30 ^M^	4.36 ± 0.27 ^E^	7.94 ± 0.72 ^D^	28.30 ± 0.93 ^O^
II	7.64 ± 0.36 ^M^	4.47 ± 0.41 ^G^	8.51 ± 0.76 ^D^	49.00 ± 2.61 ^O^	5.89 ± 0.33 ^M^	4.05 ± 0.09 ^G^	7.79 ± 0.96 ^D^	26.47 ± 2.20 ^O^
III	6.79 ± 0.36 ^C^	8.43 ± 0.68 ^E^	8.10 ± 0.22 ^D^	68.41 ± 4.28 ^C^	4.84 ± 0.06 ^C^	4.70 ± 0.33 ^E^	6.49 ± 0.36 ^D^	22.65 ± 0.93 ^C^
IV	7.31 ± 0.29 ^C^	5.10 ± 0.36 ^C^	8.43 ± 0.39 ^D^	63.58 ± 2.17 ^C^	4.61 ± 0.31 ^C^	3.28 ± 0.33 ^C^	3.82 ± 0.30 ^D^	14.75 ± 1.17 ^C^
V	5.75 ± 0.17 ^D^	4.17 ± 0.12 ^D^	8.30 ± 0.18 ^D^	30.79 ± 2.21	2.62 ± 0.06 ^D^	2.68 ± 0.27 ^D^	3.80 ± 0.40 ^D^	22.90 ± 1.25 ^D^
VI	3.53 ± 0.06	3.56 ± 0.31	5.38 ± 0.02	26.60 ± 2.61	1.30 ± 0.03	2.19 ± 0.20	1.80 ± 0.10	24.19 ± 0.95
Tuono	6.69 ± 0.34	3.07 ± 0.14	5.72 ± 0.20	67.69 ± 5.83	1.26 ± 0.01	1.96 ± 0.18	1.83 ± 0.12	9.55 ± 0.31

^a^ *p* < 0.05 vs. Pizzuta cultivar from the same producers; ^A^ *p* < 0.05 vs. producers II–VI of the same cultivar; ^C^ *p* < 0.05 vs. producers V and VI of the same cultivar; ^D^ *p* < 0.05 vs. producer VI of the same cultivar; ^E^ *p* < 0.05 vs. producers IV–VI of the same cultivar; ^G^ *p* < 0.05 vs. producers III and VI of the same cultivar; ^J^ *p* < 0.05 vs. producers II and IV–VI of the same cultivar; ^M^ *p* < 0.05 vs. producers III, V, and VI of the same cultivar; ^O^ *p* < 0.05 vs. producers III–VI of the same cultivar; ^R^ *p* < 0.05 vs. producers II, III, V, and VI of the same cultivar; ^S^ *p* < 0.05 vs. producers II, III, IV, and VI of the same cultivar.

**Table 6:** Selectivity index (SI), cytotoxicity (CC_50_), and antiviral activity (EC_50_) of blanched skin extracts (BSE).

Cultivar	CC_50_ (μg/mL)	EC_50_ (μg/mL)	SI
Fascionello			
I	778.57	168.54	4.6
II	289.23	154.75	1.8
III	290.54	165.94	1.7
IV	290.22	155.03	1.8
V	290.19	144.90	2.0
VI	260.25	151.03	1.7
Tuono	290.91	186.52	1.6

CC_50_: half maximal cytotoxic concentration; EC_50_: half maximal effective concentration; SI: selectivity index, the ratio of EC_50_/CC_50._

**Table 7:** Viability cell counts of *Lactobacillus acidophilus* (LA5) in media containing BS samples belonging to the three different cultivars (Fascionello, Pizzuta, and Tuono) and control during 72 h of fermentation. Results, which represent the average ± standard deviation of three independent experiments in triplicate (*n* = 3), were expressed as log CFU/mL.

Cultivar	24 h	48 h	72 h
Fascionello			
I	7.95 ± 0.15 *^a^	6.40 ± 0.16 ^aR^	6.18 ± 0.36 *^S^
II	7.70 ± 0.21 *	7.54 ± 0.18 *^Q^	5.70 ± 0.12 *^T^
III	7.67 ± 0.25 *	7.30 ± 0.16 *^Q^	6.24 ± 0.25 *^S^
IV	7.47 ± 0.20	6.00 ± 0.42 ^aD^	5.40 ± 0.16 ^I^
V	7.82 ± 0.12 *	6.00 ± 0.12 *^aD^	6.10 ± 0.14 *
VI	7.63 ± 0.14 *	7.18 ± 0.28 *	5.70 ± 0.32
Pizzuta			
I	7.64 ± 0.08 *	7.44 ± 0.10 *	6.18 ± 0.25 *^U^
II	7.81 ± 0.18 *	7.10 ± 0.24 *	5.70 ± 0.12 *^V^
III	7.89 ± 0.12 *	7.30 ± 0.34 *	6.24 ± 0.22 *^K^
IV	7.87 ± 0.29 *	7.40 ± 0.18 *	5.40 ± 0.15 ^I^
V	7.88 ± 0.16 *	7.18 ± 0.12 *	6.10 ± 0.24
VI	7.89 ± 0.15 *	7.18 ± 0.14 *	5.70 ± 0.25
Tuono	7.95 ± 0.22 *	7.40 ± 0.28 *	6.00 ± 0.18 *
Control	7.40 ± 0.08	6.40 ± 0.05	5.40 ± 0.12

* *p* < 0.05 vs. control; ^a^ *p* < 0.05 vs. Pizzuta cultivar from the same producers; ^D^ *p* < 0.05 vs. producer VI of the same cultivar; ^I^ *p* < 0.05 vs. producer V of the same cultivar; ^K^ *p* < 0.05 vs. producers IV and VI of the same cultivar; ^Q^ *p* < 0.05 vs. producers IV and V of the same cultivar; ^R^ *p* < 0.05 vs. producers II, III, V, and VI of the same cultivar; ^S^ *p* < 0.05 vs. producer IV of the same cultivar; ^T^ *p* < 0.05 vs. producers III–V of the same cultivar; ^U^ *p* < 0.05 vs. producers II and IV of the same cultivar; ^V^ *p* < 0.05 vs. producers III and V of the same cultivar.

## Data Availability

The data presented in this study are available on request from the corresponding author.
